# Low-Temperature (10°C) Anaerobic Digestion of Dilute Dairy Wastewater in an EGSB Bioreactor: Microbial Community Structure, Population Dynamics, and Kinetics of Methanogenic Populations

**DOI:** 10.1155/2013/346171

**Published:** 2013-09-05

**Authors:** Katarzyna Bialek, Denise Cysneiros, Vincent O'Flaherty

**Affiliations:** Microbial Ecology Laboratory, Microbiology, School of Natural Sciences and Ryan Institute, National University of Ireland, Galway, Ireland

## Abstract

The feasibility of anaerobic digestion of dairy wastewater at 10°C was investigated in a high height : diameter ratio EGSB reactor. Stable performance was observed at an applied organic loading rate (OLR) of 0.5–2 kg COD m^−3^ d^−1^ with chemical oxygen demand (COD) removal efficiencies above 85%. When applied OLR increased to values above 2 kg COD m^−3^ d^−1^, biotreatment efficiency deteriorated, with methanogenesis being the rate-limiting step. The bioreactor recovered quickly (3 days) after reduction of the OLR. qPCR results showed a reduction in the abundance of hydrogenotrophic methanogenic *Methanomicrobiales* and *Methanobacteriales* throughout the steady state period followed by a sharp increase in their numbers (111-fold) after the load shock. Specific methanogenic activity and maximum substrate utilising rate (*A*_max_) of the biomass at the end of trial indicated increased activity and preference towards hydrogenotrophic methanogenesis, which correlated well with the increased abundance of hydrogenotrophic methanogens. Acetoclastic *Methanosaeta* spp. remained at stable levels throughout the trial. However, increased apparent half-saturation constant (*K*_*m*_) at the end of the trial indicated a decrease in the specific substrate affinity for acetate of the sludge, suggesting that *Methanosaeta* spp., which have high substrate affinity, started to be outcompeted in the reactor.

## 1. Introduction

During the last decades, there has been an increased concern with the environment and the limited energy sources available. In this context, ways to treat wastewaters with energy saving methods need to be developed. Anaerobic digestion (AD) comes forward in this issue because it can treat several types of wastewaters and produce energy in the form of biogas at the same time. In order to improve the energy balance of AD, its application at low temperatures is an interesting option, especially in northern countries, where the temperatures are much lower than the optimum mesophilic temperature range of the process.

The application of low-temperature anaerobic digestion (LTAD) at temperatures of 12°C to 15°C has been studied at laboratory- and pilot-scale granular sludge-based bioreactors [1–3]. The possibility of applying the LTAD at 10°C can reduce the operation costs of the plant and improve the energy balance even further. Although LTAD at 10°C has also been studied, to date most studies have examined simple synthetic wastewaters [3]. Up to date there are only few reports describing LTAD of complex (with the presence of particulate compounds) or low-strength recalcitrant industrial waste streams [4, 5]. Therefore, the potential for low-strength anaerobic digestion of complex industrial wastewaters at 10°C remains largely unexplored.

Dairy wastewater is produced in large scale in many countries since milk and its derived products are an important part of dietary habits in various parts of the world. Dairy wastewater is defined as a complex substrate and anaerobic treatment of the dilute streams associated with milk processing can often be problematic [6]. A key future opportunity for anaerobic digestion (AD) is as a core technology for the direct treatment of low-strength, high-volume waste streams (e.g., many industrial and municipal wastewaters in temperate regions). AD is not commonly applied to such streams, due to problems of effluent quality, biomass retention, and the fact that the bioenergy harvest (plus additional external energy) must be used to heat the systems [3]. Most studies reporting treatment of dairy wastewater concentrated on (i) diversity of dairy effluents; (ii) reactor configuration; (iii) physicochemical treatment methods; and (iv) thermodynamics to enhance the treatment efficiency [6, 7]. To our knowledge, there are no reports exploring the feasibility of LTAD to digest dairy effluents at 10°C. Moreover, there is a lack of information regarding microbial composition and dynamics in such systems, which can be affected by the composition of influent wastewater [8] and environmental parameters such as temperature [1] as well as operating conditions [2]. Information about the microbial composition and dynamics in bioreactors can help to identify the optimum conditions for microorganisms growth to maximize their activity and consequently to improve reactors performance. More studies should thus be undertaken to understand the nature and function of the microbial populations involved in LTAD of dairy wastewater.

In light of this, the aim of this study was to assess the feasibility of anaerobic digestion of dilute dairy wastewater at 10°C. One of the challenges on the design of psychrophilic reactors is the retention of psychoactive biomass within low-temperature bioreactors, which is crucial for successful psychrophilic anaerobic digestion. Significant loss of granular sludge due to biomass washout has been observed at low temperatures and this problem usually intensifies as temperature decreases. Up to 43% VSS loss was observed during the operation of an expanded granular sludge bed (EGSB) operated at 10°C [9]. Since this problem was detected in a previous trial performed in an EGSB reactor (height: diameter ratio of 5.5) operated at 15°C [10], in the present study, the reactor with a higher height: diameter ratio (~10) was used during the experiment to allow the application of high upflow velocities (*V*_up_) and consequently have a better contact between substrate and biomass with reduced loss of VSS.

The process performance and microbial composition and dynamics were investigated during the course of this study. To determine the limits of stable bioreactor performance, the EGSB reactor was subjected to variable organic loading rates defined by different hydraulic retention times (HRT) and variable influent concentrations. Additionally, changes in bacterial and archaeal community structures were monitored using denaturing gradient gel electrophoresis (DGGE) and real-time PCR to investigate the influence of organic loading rates on the archaeal population dynamics during the 335-day trial. To gain further information about the microbial communities function, specific methanogenic activity (SMA) and assays to determine the maximum substrate utilising rate (*A*_max⁡_) and the Michaelis-Menten apparent half-saturation constant (*K*_*m*_) and maximum initial velocity (*V*_max⁡_) were performed. To our knowledge, this is the first study which covers so many aspects of the anaerobic digestion of a complex (dairy) wastewater at 10°C. 

## 2. Materials and Methods

### 2.1. Reactor Operation and Biomass Sampling

A glass laboratory-scale EGSB bioreactor (7.2 l working volume) treating synthetic skimmed dairy wastewater was continuously operated, at 10°C, for 335 days. The reactor's configuration was described in [11], except that in the present trial the height: diameter ratio was ~10, almost 2-fold higher than in the previous work (5.5) but still within the range of the usual EGSB's rate. The influent with the concentration of 1 kg COD m^−3^ was prepared freshly prior to each feeding. The composition of the skimmed-milk powder has been described previously in [10]. The influent was buffered with sodium bicarbonate and the pH of the bioreactor was maintained between 6.8 and 7.2 throughout the whole trial. This study is a continuation of the 430-day experiment described in [10], where the seed sludge used to inoculate the bioreactor was sourced from. The VSS concentration inoculated into the bioreactor was 17.5 g l^−1^.

The trial period was divided into six operational phases (PI–PVI; Table 1). PI (48 h HRT, days 0–146), PII (24 h HRT, days 147–230), PIII (18 h HRT, days 231–265), PIV (12 h HRT, days 266–294), PV (days 295–322) characterized by fixed HRT (12 h HRT) and increments in the OLR from 2–5 kg of COD m^−3^ d^−1^, and PVI (days 323–335) characterized by fixed HRT (12 h HRT) and return to fixed organic loading rate of 2 kg of COD m^−3^ d^−1^. The performance of the reactor was evaluated on the basis of chemical oxygen demand (COD) removal efficiency (RE), Volatile Fatty Acids (VFA) concentration in the effluent, and VFA : COD ratio; the latter was used to assess the proportion of hydrolysed substrate converted into VFAs [12].

For microbial community analysis, biomass samples were directly collected from the EGSB bioreactor on days 0, 140, 226, 255, 294, and 335. All biomass was sampled twice (2 × 50 mL) before changing operating conditions and was first mechanically disrupted by manual grinding with a pestle and mortar and diluted 4-fold with deionised and distilled water (DDW) before DNA was extracted as described previously [11]. All DNA extractions were performed in duplicate.

### 2.2. Specific Methanogenic Activity Testing

Biomass sampled from the bioreactor on day 0 and day 335 (trial conclusion) was screened for metabolic capability using specific methanogenic activity (SMA) values, performed using the pressure transducer technique [13]. Acetate (30 mM) and H_2_/CO_2_ (80 : 20, v/v) were employed to determine activity of acetoclastic and hydrogenotrophic methanogens, while propionate (30 mM), butyrate (15 mM), and ethanol (30 mM) were used as indirect methanogenic substrates to determine activity of syntrophic populations. Vials without any substrate or with the addition of N_2_/CO_2_ (80 : 20, v/v) in case of hydrogenotrophic assays served as controls. All activity assays contained 2–5 g VSS l^−1^ and were performed in triplicate. Assays on day 0 were performed at 15 and 37°C, assays on day 335 were performed at 10 and 37°C and results were expressed as mL CH_4_ g VSS^−1^ day^−1^.

### 2.3. Assessment of a Substrate Depletion Curve for the Determination of *A*_max⁡_ and *K*_*m*_

The maximum specific activity (*A*_max⁡_) and the apparent half-saturation constant (*K*_*m*_) of the sludge on acetate and H_2_/CO_2_ were determined in the beginning and end of the trial using the same serum bottles and experimental setup as for the SMA test. Instead of plotting the CH_4_ production curve, the substrate concentration in the bottles was plotted against time.

The *A*_max⁡_ was calculated from the steepest linear decline in this curve divided by the VSS in the bottle as reported by Rebac et al. [14]. The results were then expressed in g COD g VSS^−1^ d^−1^.

An integrated solution to the Michaelis-Menten equation (1) was used to determine the kinetic constants (*V*_max⁡_ (maximum initial velocity) and *K*_*m*_ (apparent half-saturation constant) from progress curves of substrate utilisation [15] as follows:

(1) $$\begin{array}{c} \frac{t}{S_{0}-S_{t}}=\frac{K_{m}}{V_{\max}}\frac{\ln\left(S_{0}/S_{t}\right)}{S_{0}-S_{t}}+\frac{1}{V_{\max}}, \end{array}$$

where *t* = time, *S*_0_ = initial substrate concentration, and *S*_*t*_ = substrate concentration at *t*. The curve ln⁡(*S*_0_/*S*_*t*_)/*S*_0_ − *S*_*t*_ against *t*/(*S*_0_ − *S*_*t*_) was plotted and the intercept and the inclination of the curve were 1/*V*_max⁡_ and *K*_*m*_/*V*_max⁡_, respectively.

### 2.4. qPCR

Real-time PCR (qPCR) analysis was performed using a LightCycler 480 instrument (Roche, Mannheim, Germany) using two methanogenic order-specific primer and probe sets: *Methanobacteriales* (MBT) and *Methanomicrobiales* (MMB), representing hydrogenotrophic methanogens and two methanogenic family-specific primer and probe sets: *Methanosarcinaceae* (Msc) and *Methanosaetaceae* (Mst), as described previously [10, 11]. All DNA templates were analysed in duplicate. 

Because the other hydrogenotrophic methanogens *Methanopyrales* and *Methanococcales* (MCC) members are not likely to be present in anaerobic bioreactors due to their extremely high growth temperature (>80°C) and high-salt requirements (0.3–9.4% (w/v) NaCl), respectively [16], these two orders were left out of consideration in this study. 

### 2.5. Archaeal and Bacterial DGGE

Archaeal and bacterial 16S rRNA genes amplification, touchdown PCR, PCR products purification, sequencing, sequencing alignment, and phylogenetic analyses were performed as described previously [11]. Unweighted Pair Group Method with Arithmetic mean (UPGMA) was selected to perform statistical analysis of the DGGE profiles as described previously [10]. All nucleotide sequence data reported in this study were deposited in the GenBank database under accession numbers ARC (A3–A5): JQ730820–JQ730822 and BAC (B1–B25): JQ730824–JQ730848.

### 2.6. Analytical Analysis

COD analysis was performed according to standard methods (APHA, 2005). VFAs analysis was performed in a Varian Saturn 2000 GC/MS system, with CombiPAL autosampler (Varian Inc., Walnut Creek, CA) as described previously [11]. Biogas composition in the headspace of serum bottles used for the SMA and kinetic tests was determined. The analysis was performed by gas chromatography (Varian) using a glass column (1.8 m × 6 mm outer diameter × 4 mm inner diameter) packed with Porapak Q 100–120 mesh in a Philips PYE-Unicam Series 304 chromatograph fitted with a gas sampling port and a flame ionisation detector. The column temperature was maintained at 35°C. The injection port and detector temperatures were 105°C and 100°C, respectively. N_2_ was the carrier gas at a flow rate of 25 mL min^−1^. 

## 3. Results and Discussion

### 3.1. Bioreactor Performance

**Figure 1:**
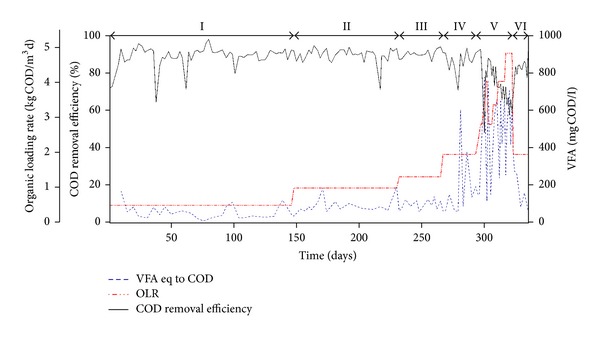
Chemical Oxygen Demand (COD) removal efficiency (RE), Volatile Fatty Acids (VFA concentrations; presented as sum of acetic-, butyric-, iso-butyric-, propionic-, valeric-, and iso-valeric), and organic loading rate (OLR) applied to the expanded granular sludge bed (EGSB).

Figure 1 illustrates the COD removal efficiency (RE) profiles and effluent VFA concentrations associated with the EGSB bioreactor during the trial. The operating parameters and performance are summarized in Table 1. The EGSB reactor demonstrated >85% COD RE and effluent concentrations of <140 mg COD L^−1^ VFA during steady-state operation of phases PI–PIV. After the OLR increased to 4.1 kg COD m^−3^ d^−1^, during PV, the COD RE suddenly dropped to 48% and VFA concentrations peaked at >770 mg COD L^−1^. At this point, VFAs represented ~60% of the remaining COD in the effluent, indicating that methanogenesis was rate-limiting at this applied OLR. This is an interesting result since hydrolysis was the rate-limiting step in the same reactor operated at 15°C [10]. Other researchers have found that the rate-limiting step of the process was hydrolysis at the operational temperature of 15°C but methanogenesis was rate-limiting at 10°C [17]. Once the OLR was reduced to 2.9 kg COD m^−3^ d^−1^, the COD RE recovered (>80%) and the VFA effluent concentrations dropped to <140 mg COD L^−1^. However, when gradual increases in the OLR (up to 5 kg COD m^−3^ d^−1^) were applied, a gradual drop in the COD RE (~60%) and fluctuations in the effluent VFA concentrations (>760 mg COD L^−1^) were observed. At this stage, VFAs accounted for 76% of the COD present in the effluent, again indicating that methanogenesis was rate-limiting at a higher OLR. During PVI (characterized by return to fixed HRT of 12 h and return to fixed OLR of 2 kg COD m^−3^ d^−1^), a rapid improvement was observed in the bioprocess performance, with mean COD RE exceeding 84% and a decrease in effluent VFA concentrations to <60 mg COD L^−1^ by the end of trial.

To overcome the problem of biomass washout at low temperatures, an EGSB with a high height: diameter ratio (~10) was used in this trial. The increased height: diameter ratio allowed the application of high *V*_up_ (Table 1) while successfully retained most of the sludge in the reactor. While previous work at 10°C reported up to 43% VSS loss from an EGSB operated at similar *V*_up_ with a height: diameter ratio of 5.5 [9], a relatively low VSS loss was observed in this trial (17%) at the end of the operation time (day 335). 

### 3.2. Real-Time PCR of Archaeal Populations

**Figure 2:**
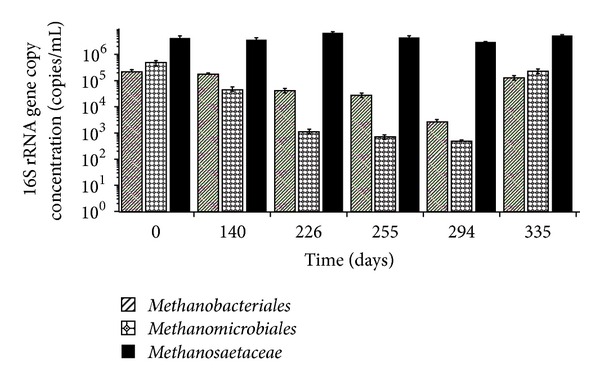
Absolute quantification of the 16S rRNA gene concentration of the methanogenic/archaeal populations during psychrophilic (10°C) EGSB bioreactor operation.

The real-time PCR results show clearly a gradual decrease in the 16S rRNA gene concentrations of the hydrogenotrophic groups *Methanomicrobiales* (MMB) and *Methanobacteriales* (MBT) from PI to PIV (Figure 2). At the beginning of trial (PI; day 0), MMB accounted for 9.9% (4.9 × 10^5^ copies/mL) and MBT accounted for 4.4% (2.2 × 10^5^ copies/mL) of the total methanogenic 16S rRNA gene concentration (Figure 2). With the decrease in HRT from 48 to 12 h (PI–PIV), a 975-fold reduction in the concentrations of MMB (16S rRNA gene concentration of 5.0 × 10^2^ copies/mL) and an 80-fold reduction in the numbers of MBT were observed (16S rRNA gene concentration of 2.7 × 10^3^ copies/mL). Although a significant decrease in abundance of those two hydrogenotrophic groups occurred, there was no deterioration in the bioreactor performance during the corresponding periods even though the OLR was 6-fold higher (Figure 1).

Interestingly, at the end of the trial (day 335, PVI) both MMB and MBT numbers recovered, with a marked 456-fold (16S rRNA gene concentration of 2.3 × 10^5^ copies/mL) and 47-fold (16S rRNA gene concentration of 1.3 × 10^5^ copies/mL) increase, respectively. This could be attributed to a stress response induced by the fast increase in the OLR during PV (increase in OLR from 2 to 5 kg COD m^−3^ d^−1^), before its reduction during PVI (OLR back to 2 kg COD m^−3^ d^−1^). Increased OLR along with low temperatures has previously been shown to cause proliferation of *Methanomicrobiales* sp. due to stress response [2]. This can occur because higher OLRs lead to higher in-reactor VFA concentrations, as it was the case during PV, which has been reported to cause an increase in the syntrophic acetate degradation pathway (to CH_4_ via H_2_/CO_2_), especially at low temperatures [18]. This increase in the abundance of *Methanomicrobiales* during mesophilic and thermophilic reactor operation has been previously reported [11, 19] but to our knowledge, this is the first work reporting this fact at psychrophilic (10°C) conditions. 

The acetoclastic family *Methanosaetaceae* (Mst) was the most abundant and stable group during the whole trial, indicating that OLR changes applied during the trial did not influence or perturb this community (Figure 2). The 16S rRNA gene concentration of *Methanosaetaceae* accounted for 85.6% (4.2 × 10^6^ copies/mL) and 93.7% (5.3 × 10^6^ copies/mL) of the total methanogenic population at the beginning (PI; day 0) and at the end (PVI; day 335) of the trial, respectively. These results indicate that this acetoclastic family was an important member of the methanogenic community and may be retained within anaerobic biofilms during cold (10°C) bioreactors operation [20]. 

The acetoclastic family, *Methanosarcinaceae* (Msc), was not detected (i.e., <5.63 × 10^1^ copies/*μ*L) in the bioreactor throughout the trial and therefore our qPCR results showed that the methanogenic acetate conversion could be attributed to the members of *Methanosaetaceae* (Mst) family. Under steady operational conditions, during PI–PIV, the low prevailing residual acetate concentrations (65 ± 31 mg COD L^−1^; Figure 1) coupled with low temperature contributed to the suppression of *Methanosarcina* by *Methanosaeta* as the latter is known to have high substrate affinity and therefore is a better scavenger for acetate than the former [21]. Although the residual acetate concentrations sporadically reached higher values than the threshold value required to support the growth of *Methanosarcina* [22] during PV (peak at 670 mg COD L^−1^), this group remained below the detection limit. It is known that acetoclastic methanogens have very low growth rates, which result in doubling times of several days or more [23]; therefore, the intermittent periods of high acetate peak did not allow time for this group to grow.

### 3.3. Specific Methanogenic Activity (SMA) and Kinetic of Methanogenic Populations

SMA results indicated the mesophilic nature of the inoculum (day 0) and biomass at the end of trial (day 335), exhibiting higher activity at 37°C than at 10 and 15°C, for all substrates tested (Table 2). However, as was previously observed during cold cultivation [24] our results indicate that mesophiles may metabolise and grow under suboptimal temperatures [25]. The maximum substrate utilization rates (*A*_max⁡_) of the sludge at 37°C (Table 3) support the SMA results and agree with previous work which showed the strong effect of temperature on the activity of methanogens [26]. 

Assays carried out at the beginning of the trial (day 0) showed that the biomass displayed a preference for hydrogenotrophic methanogenesis at 37 and 15°C. By the end of trial (day 335) SMA values against H_2_/CO_2_ at 37°C were almost 2-fold higher than those determined at the beginning (day 0) revealing continued preference towards hydrogenotrophic methanogenesis. However, metabolic activity determined by the end of trial (day 335) at 10°C (the same as bioreactor operational temperature throughout the trial) indicated equal capacity for acetate-mediated and H_2_/CO_2_-mediated methanogenesis as it is shown by the similar SMA and *A*_max⁡_ values (Tables 2 and 3).

As mentioned earlier, the 16S rRNA gene concentration of *Methanosaetaceae* showed only a slight increase from the beginning to the end of the trial (Table 2), suggesting that there was no major change in the abundance of this group. However, the SMA indicated that the metabolic activity against acetate at 37°C at the end of trial was lower (257 ± 6; day 335) than at the beginning (366 ± 26; day 0) (Table 2). The data for the maximum specific substrate utilization rate (*A*_max⁡_) of the sludge (Table 3) agree well with the SMA results and show a reduction of 26% in the *A*_max⁡_ on acetate at 37°C from the beginning to the end of trial. It is likely that even though *Methanosaeta* were present in the reactor, they were suppressed during PV due to the increased acetate concentrations since their substrate affinity is high and therefore, they require low acetate concentrations to grow. This hypothesis is supported by the decrease in the substrate affinity of the sludge on acetate at 37°C, characterized by the 45% increase in the apparent half-saturation constant (*K*_*m*_) at the end of the trial (Table 3). This result indicates a shift in the microbial community in the sludge, where microorganisms with lower substrate affinity started to outcompete *Methanosaeta*. Since the other major acetotrophic methanogens *Methanosarcinaceae* were not detected in the sludge at the end of the trial, possibly syntrophic oxidation of acetate started to play an important role in the reactor when VFA concentrations were high during PV, a condition which has been shown to favour syntrophic acetate oxidation [27]. The 2-fold and 7-fold increase in the SMA and *A*_max⁡_ on H_2_/CO_2_ at 37°C, respectively (Tables 2 and 3), at the end of the trial supports this hypothesis as does the fact that the numbers of hydrogenotrophic methanogens increased during the disturbance period (PV). 

Looking at the kinetics study done with sludge grown for 335 days at 10°C, it was observed that the *A*_max⁡_ on both acetate and at H_2_/CO_2_ at 10°C (0.246 and 0.199 g COD g VSS^−1^ d^−1^, resp.; Table 3) were in the same order of magnitude of the *A*_max⁡_ reported in previous work at the same temperature, which were considered to be high (0.331 and 0.296 g COD g VSS^−1^ d^−1^, resp.; [14]). The *K*_*m*_ of the sludge on H_2_/CO_2_ at 10°C (0.008 g COD g VSS^−1^ d^−1^ or 11.4 *μ*M; Table 3) was higher than the *K*_*m*_ of a mixed community from sediments grown at 9°C (7.1 *μ*M; [28]). On acetate, the *K*_*m*_ of the sludge (0.006 g COD L^−1^) was 10-fold lower than the *K*_*m*_ value of a granular sludge at 10°C presented by Rebac et al. [14] (0.058 g COD L^−1^) but this could have been caused by the conditions of the test. The latter study used an EGSB reactor operated in batch mode for the kinetic assay, which certainly improved substrate-sludge contact and increased the *K*_*m*_ value. In summary, the results of the kinetic assays performed at 10°C correlated well with previously reported data and showed that the sludge grown at 10°C in the EGSB presented here had a high substrate utilization rate (*A*_max⁡_) at 10°C on both methanogenic substrates.

### 3.4. Archaeal and Bacterial DGGE and Phylogenetic Analysis

Archaeal and bacterial community fingerprinting was carried out using denaturing gradient gel electrophoresis (DGGE). Extracted DNA samples were analyzed to investigate changes in methanogenic populations with respect to the HRT and OLR changes throughout the 10°C trial. 

**Figure 3:**
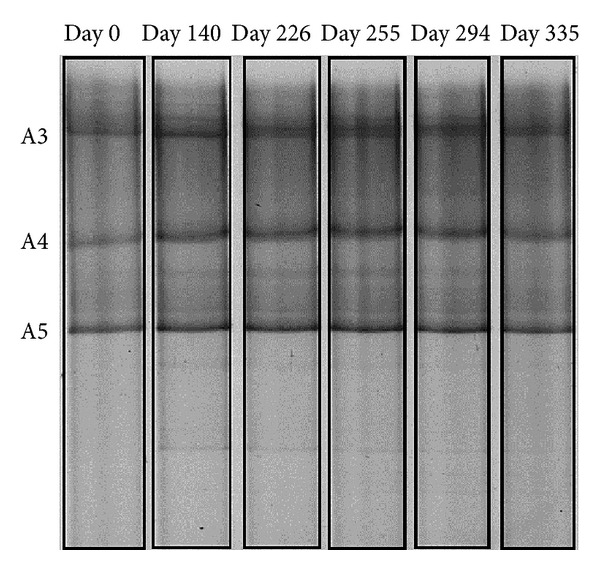
Denaturing gradient gel electrophoresis (DGGE) profiles of temporal archaeal 16S rRNA genes from EGSB biomass.

In the archaeal DGGE gel, three bands (A3, A4, A5) were visible throughout the whole trial (Figure 3) and were possible to retrieve and sequence (Figure 5(b)). Band A3 was closely related to hydrogen-utilizing *Methanocorpusculum* like clones (*M. bavaricum, M. parvum, M. labreanum, M. sinense*) with 99% sequence similarity. Similar clones were previously observed during the cold adaptation of anaerobic granular and flocculant sludge [20]. Band A4 showed 99% similarity to hydrogen-utilizing *Methanospirillum hungatei* and was detected in all 10°C biomass samples. This organism has been previously observed during psychrophilic (15°C) anaerobic digestion in EGSB bioreactors [10]. Band A5 was closely related to acetoclastic *Methanosaeta concilii* (100% similarity) and was detected throughout the whole trial. 

**Figure 4:**
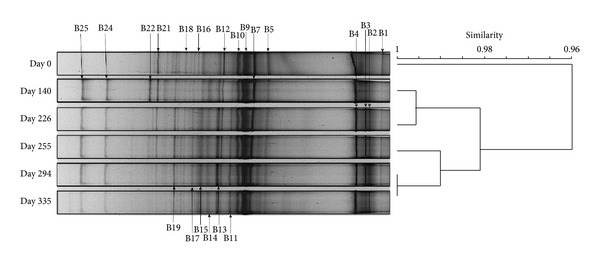
Unweighted Pair Group Method with Arithmetic Mean (UPGMA) cluster analysis of the 16S rRNA gene fragments generated from bacterial denaturing gradient gel electrophoresis (DGGE) profiles of EGSB biomass. Similarity calculated by Sørensons (Bray-Curtis) distance measurement.

The UPGMA cluster analysis of bacterial DGGE profiles revealed >96% similarity of all DGGE profiles of the bacterial populations within the whole trial (PI–PVI; EGSB: 0, 140, 226, 255, 294, 335; Figure 4) suggesting resistance of the bacterial community composition [29] despite changes in the HRT and OLR applied during the trial. 

**Figure 5:**
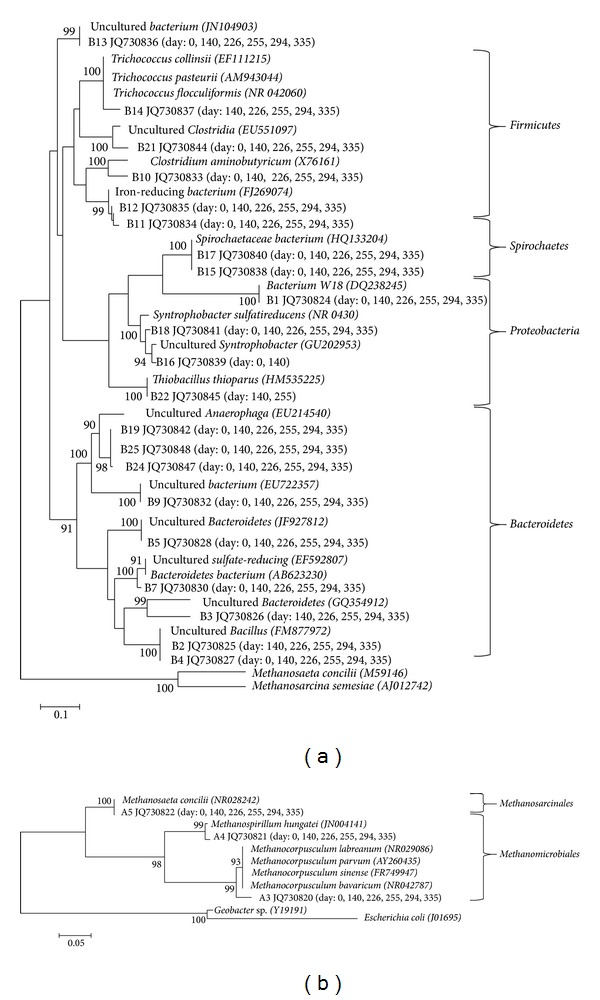
Neighbor joining tree illustrating the phylogenetic affiliations of the 16S rRNA gene sequences obtained from (a) bacterial DGGE bands and (b) archaeal DGGE. Bioreactor biomass samples taken on various days and containing the respective bands are given in parenthesis.

Partial 16S gene sequences from 20 out of total 21 excised bacterial DGGE bands grouped within four phyla: *Firmicutes, Proteobacteria, Spirochaetes,* and *Bacteroidetes* (Figure 5(a)).

The phylum *Firmicutes* was represented by five bands B10, B11, B12, B14, and B21, which were mostly present in all biomass samples (Figure 5(a)). Information on the identity and potential role of some of these bacteria could be tentatively inferred from the phylogenetic analysis. B10 showed 98% similarity to *Clostridium aminobutyricum* and was present in the bioreactor during the whole 10°C trial even though its optimum temperature growth was reported to be in the mesophilic range [30]. This is not surprising since the sludge in the reactor kept its mesophilic nature as shown by the SMA and *A*_max⁡_ results presented previously. B14 was apparent during the whole trial (except day 0) and shared 99% similarity with *Trichococcus flocculiformis, Trichococcus collinsii,* and *Trichococcus pasteurii*, aerotolerant, fermentative organisms growing with glucose, sucrose, and lactose to produce lactate, acetate, formate, and other acids [31]. 

The phylum *Proteobacteria* was represented by four bands (B1, B16, B18, B22). Although B16 and B18 were not closely related they shared 94–95% similarity to *Syntrophobacter sulfatireducens*, propionate-oxidizing bacterium [32]. Growth of this organism was observed between 20 and 48°C [33] but it was previously reported during psychrophilic (15°C) anaerobic bioreactor operation [10]. Propionate is fermented syntrophically to acetate and CO_2_ in the presence of hydrogen-/or formate-utilizing methanogens like *Methanospirillum hungatei* [33], which has been identified as band A4 retrieved from archaeal DGGE (Figures 3 and 5(b)). The presence of *Syntrophobacter sulfatireducens*-like clones during the whole trial in the EGSB reactor putatively suggests that it was important during propionate oxidation, which has been identified as the rate-limiting step during LTAD [25]. The emergence of psychrophilic propionate-utilizing populations was correlated with propionotrophic activity during long-term operation under low-temperature conditions [20]. B22 showed 99% similarity to *Thiobacillus thioparus*, thiosulphate oxidizing bacterium commonly used for Total Reduced Sulphur biofiltration technology [34] and was apparent at day 140 and 255. 

The phylum *Spirochaetes* was represented by two bands (B15 and B17), which shared 99% similarity to *Spirochaetaceae bacterium* and were present in all 10°C biomass samples. Although *Spirochaetes* were reported to be numerically important in psychoactive consortia [20], their function is not clear. 

The phylum *Bacteroidetes* was represented by nine bands (B2, B3, B4, B5, B7, B9, B19, B24, B25), which were present in all 10°C biomass samples, but that were mostly affiliated to uncultured organisms and their function remains unclear. 

Even though molecular techniques allowed a better understanding about the structure and dynamics of microbial communities in bioreactors, much work still needs to be done to understand the role these major bacterial and archaeal groups play. Therefore, future studies should address the functional characteristics of these groups in the reactors. This will provide important information which will allow the optimization of the process and consequently broad the application of AD. This is a key consideration for LTAD in particular, as it affects the range and types of feedstocks and wastewaters to which it can be applied.

## 4. Conclusions

Anaerobic digestion of dilute dairy wastewater was possible at 10°C at applied OLRs up to 2 kg COD m^−3^ d^−1^ with COD RE above 84%. At OLRs above this level, COD RE dropped and VFAs accumulated in the reactor with methanogenesis being the rate-limiting of the process. The reactor recovered quickly (3 days) after the load shock, once the OLR was reduced to 2 kg COD m^−3^ d^−1^. A high height: diameter ratio (10) of the EGSB was important to retain the biomass in the reactor through a long operation period at 10°C. 

During steady state conditions, acetoclastic methanogens *Methanosaeta* spp. appeared to be the dominant group while no *Methanosarcinaceae* were detected in the reactor and the numbers of hydrogenotrophic methanogenic groups *Methanomicrobiales* and *Methanobacteriales* decreased through time. During the period of reactor's disturbance, the numbers of hydrogenotrophic methanogens sharply increased as a stress response induced by the applied shock load. An increase in the SMA and *A*_max⁡_ on H_2_/CO_2_ and a decrease in the SMA and *A*_max⁡_ on acetate at the end of the trial, along with the increased numbers of hydrogenotrophic methanogens, suggested a shift in the pathway of methane formation from acetotrophic to syntrophic oxidation of acetate at high OLR. A decrease in the substrate affinity, characterized by an increase in the *K*_*m*_ on acetate at the end of the trial indicated that *Methanosaeta* spp. started to be outcompeted by other microorganisms with lower substrate affinity due to the high OLR applied.

## Figures and Tables

**Table 1:** Operational and performance characteristics during phases I to VI of the bioreactor operation.

Phases	PI	PII	PIII	PIV	PV	PVI
Days	0–146	147–230	231–265	266–294	295–322	323–335
HRT^a^	48	24	18	12	12	12
OLR^b^	0.5	1.0	1.3	2.0	2.0/5.0	2.0
SLR^d^	0.03	n.d.	n.d.	n.d.	n.d.	0.14
*Q* ^ e^	0.0036	0.0072	0.0096	0.0144	0.0144	0.0144
*V*_up_^f^	1.93	1.95	1.96	1.99	1.99	1.99
*C*_*s*_^g^	1.0	1.0	1.0	1.0	1.0/2.5	1.0
CODRE^c^	88 ± 6	89 ± 4	90 ± 2	86 ± 6	74 ± 11	84 ± 4
CH_4theo_^h^	0.151	0.277	0.328	0.706	1.310/3.276	0.806
TVFA^i^	54	91	97	178	466	184
VFA: COD ratio	0.45	0.83	0.97	1.00	0.76	1.00

^a^Hydraulic retention time (h).

^b^Organic loading rate (kg COD m^−3^ d^−1^).

^c^Chemical oxygen demand removal efficiency (%), where values are the phases mean (±s.d.).

^d^Sludge loading rate (kg COD kg [VSS]^−1^ d^−1^); n.d.: not determined.

^e^ *Q* flow rate (m^3^ d^−1^).

^f^ *V*_up_ upflow velocity (m h^−1^).

^g^C_s_ substrate concentration (kg COD m^−3^).

^h^CH_4theo_ theoretical methane production (l CH_4_ d^−1^). Calculated stoichiometrically assuming that 1 g COD removed produced 0.350 L of CH_4_.

^i^Total Volatile Fatty Acids (mg COD L^−1^).

**Table 2:** Specific methanogenic activity (SMA) profiles against direct and indirect methanogenic substrates expressed as mL CH_4_ g VSS^−1^ day^−1^ and the absolute quantification of 16S rRNA gene copy concentration expressed in copies/mL, of day 0 and day 335 biomass.

	*T* (°C)	Specific methanogenic activity (mL CH_4_ g VSS^−1^ day^−1^)					Absolute quantification by qPCR	
		SMA Substrate					16S rRNA gene copy concentration (copies/mL)	
		Acetate	H_2_/CO_2_	Ethanol	Propionate	Butyrate	Mst	MBT + MMB
Biomass day 0	37	366 ± 26	422 ± 21	336 ± 27	203 ± 20	293 ± 19*	4.2 × 10^6^	7.1 × 10^5^
	15	87 ± 10	91 ± 4	85 ± 3	59 ± 7	59 ± 2	4.2 × 10^6^	7.1 × 10^5^

Biomass day 335	37	257 ± 6	774 ± 42	425 ± 21*	140 ± 4	222 ± 13	5.4 × 10^6^	3.6 × 10^5^
	10	38 ± 4	37 ± 4	29 ± 5	7 ± 0	22 ± 3	5.4 × 10^6^	3.6 × 10^5^

All SMA values are the mean of triplicates (std. error; *n* = 3), except *where values are the mean of duplicates (std. error; *n* = 2). Absolute quantification of 16S rRNA gene copy concentration of the groups: Mst (*Methanosaetaceae*), MBT (*Methanobacteriales*) and MMB (*Methanomicrobiales*).

**Table 3:** Maximum substrate utilising rate, apparent half-saturation constant, and maximum initial (utilising) velocity changes of granular sludge cultivated at 10°C in the EGSB during 335 days for acetate and H_2_/CO_2_.

	*T* (°C)	Substrate					
		H_2_/CO_2_			Acetate		
		*A*_max⁡_^a^	*K*_*m*_^b^	*V*_max⁡_^c^	*A*_max⁡_^a^	*K*_*m*_^b^	*V*_max⁡_^c^
Biomass day 0	37	1.230 (±0.148)	0.007 (±0.002)	0.580 (±0.046)	2.208 (±0.647)	0.088 (±0.032)	0.722 (±0.265)
	15	0.172 (±0.015)	0.020 (±0.004)	2.058 (±0.674)	0.419 (±0.103)	0.022 (±0.007)	0.265 (±0.125)

Biomass day 355	37	8.526 (±0.525)	0.558 (±0.419)	23.818 (±8.635)	1.638 (±0.746)	0.163 (±0.024)	2.422 (±0.336)
	10	0.199 (±0.071)	0.008 (±0.004)	1.276 (±0.004)	0.246 (±0.037)	0.006 (±0.003)	0.413 (±0.303)

^a^Maximum substrate utilising rate (g COD g VSS^−1^ d^−1^).

^b^Apparent half-saturation constant (g COD L^−1^).

^c^Maximum initial velocity (g COD L^−1^ d^−1^).
